# Mechanochemistry of Chitosan-Coated Zinc Sulfide (ZnS) Nanocrystals for Bio-imaging Applications

**DOI:** 10.1186/s11671-017-2103-z

**Published:** 2017-05-04

**Authors:** Zdenka Bujňáková, Erika Dutková, Martin Kello, Ján Mojžiš, Matej Baláž, Peter Baláž, Oleh Shpotyuk

**Affiliations:** 1 Institute of Geotechnics, Slovak Academy of Sciences, Watsonova 45, 04001 Košice, Slovakia; 2 Faculty of Medicine, P.J.Šafárik University, Trieda SNP1, 04011 Košice, Slovakia; 3 Vlokh Institute of Physical Optics, 23 Dragomanov, 79005 Lviv, Ukraine; 4 Institute of Physics, Jan Dlugosz University, 13/15, Armii Krajowej al., 42200 Czestochowa, Poland

**Keywords:** Zinc sulfide, Chitosan, Bio-imaging, Nanosuspension, Mechanochemistry

## Abstract

The ZnS nanocrystals were prepared in chitosan solution (0.1 wt.%) using a wet ultra-fine milling. The obtained suspension was stable and reached high value of zeta potential (+57 mV). The changes in FTIR spectrum confirmed the successful surface coating of ZnS nanoparticles by chitosan. The prepared ZnS nanocrystals possessed interesting optical properties verified in vitro. Four cancer cells were selected (CaCo-2, HCT116, HeLa, and MCF-7), and after their treatment with the nanosuspension, the distribution of ZnS in the cells was studied using a fluorescence microscope. The particles were clearly seen; they passed through the cell membrane and accumulated in cytosol. The biological activity of the cells was not influenced by nanoparticles, they did not cause cell death, and only the granularity of cells was increased as a consequence of cellular uptake. These results confirm the potential of ZnS nanocrystals using in bio-imaging applications.

## Background

Zinc sulfide (ZnS) has been one of the most studied semiconductor materials, because of interesting properties, which can be applied in optoelectronic, photocatalytical, and biomedical field [1]. Its transition from bulk- to nanosized particles has brought forth some drastic changes in its properties, mainly in the optical ones [2]. Nanoparticles offer opportunities to become a system for targeted drug delivery as well as imaging agent, thanks to their multi-functionalization [3]. Several studies dealing with the biomedical application of ZnS have been published recently [4–8]. In these papers, as well as in plenty of others, the results show that the prepared nanoparticles based on zinc sulfide exhibit high quantum yield, which can be utilized in fluorescence images for the better resolution of the appropriate biological structures.

When using inorganic nanoparticles for bio-imaging applications, it is necessary to cover them by a biocompatible, organic material to become acceptable for bio-systems and for the study of possible changes in cells, tissues, or organs. However, it is a natural property of nanoparticles to coagulate and agglomerate which, is a result of their large surface area. From this point of view, it is important to ensure their water dispersibility [5] and stability [9]. This can be achieved by modification of their surface using an appropriate capping agent, e.g., polymer [10, 11], surfactant [9, 12, 13], silica layer [14], lipid layer [15], and amino acids [16]. The covering of nanoparticle surface by chitosan was also used in many examples [17–19]. Chitosan is a nontoxic, polycationic polymer that has been broadly used in pharmaceuticals, drug carriers, and delivery systems. The capping of ZnS nanoparticles with this polymer was also described [20, 21].

ZnS or ZnS-capped chitosan nanoparticles were prepared by several methods, e.g., γ-radiation [20], colloidal synthesis [21], or co-precipitation [22]. The wet mechanochemical approach was also successfully applied for the preparation of nanoparticles covered by biocompatible material [11, 13, 16]. Binary InAs/ZnS system was covered by chitosan as well [17]. The obtained nanosuspensions were stable for a long time without formation of aggregates. The preparation of nanosuspension containing pure ZnS and chitosan as capping agent by mechanochemical route was not reported until now. Therefore, in this paper, it was prepared by wet milling using a circulation mill. Using this method, the nanoparticles of ZnS were well dispersed in water solution based on chitosan. The properties of the obtained suspension were determined using zeta potential measurement, particle size distribution, and FTIR technique. Moreover, its biocompatibility and bio-imaging properties were confirmed in vitro on four selected cancer cell lines.

## Methods

The nanosuspension was prepared in a laboratory circulation mill MiniCer (Netzsch, Germany). Four grams of ZnS nanocrystalline sample (prepared according to procedure described in [23]) was subjected to wet milling process in the presence of 300 mL chitosan (high molecular weight, *M*(*w*) = 310–375 kDa, >75% deacetylated, Sigma-Aldrich, USA) water solution (0.1 wt.%) for the duration of 120 min at the milling speed of 3500 rpm. The mill was loaded with yttrium-stabilized ZrO_2_ balls, 0.6 mm in diameter. The resulting nanoparticle suspension was centrifuged at 8000 rpm. Afterwards, the nanosuspension was characterized and stored in refrigerator (4 °C).

The particle size distribution was measured by photon cross-correlation spectroscopy using a Nanophox particle size analyzer (Sympatec, Germany). A portion of each nanosuspension was diluted with the stabilizer-containing solution to achieve a suitable concentration for the measurement. This analysis was performed using a dispersant refractive index of 1.33. The measurements were repeated three times for each sample.

The zeta potential (ZP) was measured using a Zetasizer Nano ZS (Malvern, Great Britain). The equipment measures the electrophoretic mobility of the particles, which is converted to the zeta potential by using the Helmholtz–Smoluchowski equation built into the Malvern zetasizer software. The zeta potential was measured in the original dispersion medium, and the measurements were repeated three times with at least 12 subruns for each sample. The average values were denoted.

The FTIR spectra were recorded using a Tensor 29 infrared spectrometer (Bruker, Germany) using the ATR method.

The optical spectra were recorded using a UV–Vis spectrophotometer Helios Gamma (Thermo Electron Corporation, Great Britain) in the range 200–800 nm.

The photoluminescence (PL) spectra at a room temperature were acquired at the right angle on a photon counting spectrofluorometer PC1 (ISS, USA) with an excitation wavelength of 365 nm. A 300-W xenon lamp was used as excitation source. The emission was collected in a 25 cm monochromator with a resolution of 0.1 nm equipped with a photomultiplier.

The human cancer cell lines HCT116 (human colorectal carcinoma) and HeLa (human cervical adenocarcinoma) were cultured in RPMI 1640 medium (Biosera, Kansas City, MO, USA). CaCo-2 (human colorectal adenocarcinoma) and MCF-7 (human breast adenocarcinoma) cell lines were maintained in a growth medium consisting of high-glucose Dulbecco’s modified Eagle’s medium with sodium pyruvate (GE Healthcare, Piscataway, NJ, USA). Both media were supplemented with a 10% fetal bovine serum (FBS), penicillin (100 IU/mL), and streptomycin (100 μg/mL) (all Invitrogen, Carlsbad, CA, USA) in an atmosphere containing 5% CO_2_ in a humidified air at 37 °C. The cell viability, estimated by the trypan blue exclusion, was greater than 95% before each experiment.

The metabolic activity colorimetric assay (MTS) was used to determine the effects of ZnS nanosuspension (*c*_Zn_ = 1–10 μg/mL) on the metabolic activity of different cell lines. After 72 h of incubation, 10 μL of MTS (Promega, Madison, WI, USA) was added to each well according to the CellTiter 96® AQueous One Solution Cell Proliferation Assay protocol. After minimum 1 h incubation, the absorbance was measured at 490 nm using the automated Cytation™ 3 Cell Imaging Multi-Mode Reader (Biotek, Winooski, VT, USA). The absorbance of the control wells was taken as 100%, and the results were expressed as a percentage of the control. All experiments were performed in triplicate.

For the flow cytometry analyses of cell granularity, the cells were seeded at a density of 3 × 10^4^ in Petri dishes (Sarstedt, Germany). Twenty-four hours after cell seeding, the cells were treated with ZnS nanosuspension (*c*_Zn_ = 0.5 μg/mL) for 72 h, washed two times with 1× PBS (Sigma-Aldrich, Great Britain) and harvested by trypsinization. The uptake of the nanoparticles by the different cell lines was analyzed through granularity (side scatter of light (SSC-H) vs. forward scatter of light (FSC-H)) changes on FACSCalibur flow cytometer (Becton Dickinson, USA).

For cell imaging analyses, the cells (6 × 10^4^) were seeded on six-well plates (Sarstedt, Germany) and cultivated for 24 h in a complete medium with 10% FBS. Afterwards, the cells were treated with ZnS nanosuspension (*c*_Zn_ = 1 μg/mL) for 72 h. At the end of the incubation time, the cells were washed twice in 1× PBS, fixed with 4% paraformaldehyde and permeabilized with 90% methanol (Ites, Slovakia) for 20 min on ice. The nuclei were stained with SlowFade® Gold antifade reagent with 4′,6-diaminidino-2-phenyl-indole dihydrochloride (DAPI) (Invitrogen). The slides were analyzed using Cytation™ 3 Cell Imaging Multi-Mode Reader (Biotek).

## Results and Discussion

### Characterization of ZnS Nanocrystals

The ZnS nanocrystals were synthesized by the mechanochemical route from zinc acetate and sodium sulfide precursors as was described for the first time in our previous work [23]. The XRD analysis confirmed the presence of both cubic sphalerite (β-ZnS) and hexagonal wurtzite (α-ZnS) phases.

The structure of ZnS nanocrystals with the crystallite size of 2–4 nm was clearly identified by Williamson–Hall analysis and Warren–Averbach method, which was in good accordance with HRTEM analysis. The nanocrystal aggregate formation, and the surface uniformity and homogeneity were well documented. The UV–Vis absorption spectrum showed a blue shift compared with the bulk ZnS indicating its quantum confinement. The more detailed structure and surface as well as optical properties of the mechanochemically synthesized ZnS nanoparticles were studied in paper [24].

The micro-Raman and micro-photoluminescence spectra with the calculated quantum yield of ZnS nanocrystals were published in our previous paper [25]. The Raman spectrum of ZnS showed one intensive peak, centered at 346 cm^−1^, and a weak peak, centered at 690 cm^−1^, associated with the first-order longitudinal optimal photon (1LO) and second (2LO) vibrational mode of ZnS, respectively. The micro-photoluminescence spectrum of ZnS comprised most of the visible spectrum with quantum yield of 2.5% at room temperature showing the role of holes/electron interactions.

### Preparation of Chitosan-Coated ZnS Nanocrystals

**Fig. 1:**
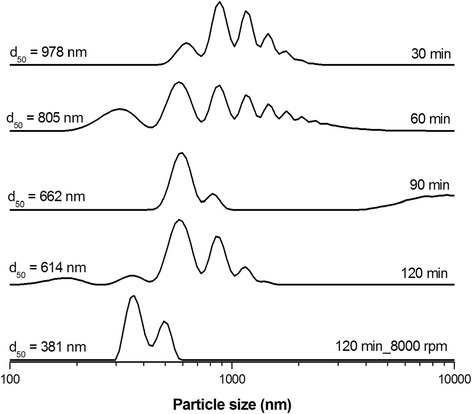
Evolution of particle size distribution during the preparation of chitosan-coated ZnS nanosuspension using wet ultra-fine milling. Milling time, revolutions of centrifugation, and average particle size *d*_50_ are described

In order to obtain well-dispersed ZnS nanocrystals, the wet ultra-fine milling in chitosan water solution (0.1 wt.%) was performed, thus resulting in the preparation of nanosuspension. The evolution of the particle size distribution during the milling process is shown in Fig. 1. As can be seen, the average particle size, *d*_50_, of obtained suspension was gradually decreasing with the increasing milling time (from 987 nm after 30 min to 614 nm after 120 min). In all cases, the distributions were of polymodal shape. By subsequent centrifugation of the sample milled for 120 min at 8000 rpm, it was possible to affect the parameter *d*_50_ further and the obtained distribution curve had bimodal shape. The largest particles with the sizes of micrometer range disappeared, and only nanosized particles with the average size, *d*_50_ = 381 nm, remained in suspension. It was not possible to obtain unimodal particle size distribution by subsequent increasing of centrifugal force. Such prepared ZnS-chitosan suspension was stable, and the particles did not settle down.

**Fig. 2:**
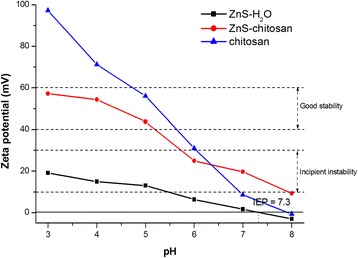
Zeta potential versus pH dependence for pure chitosan and ZnS nanocrystals measured in water and chitosan

The zeta potential (ZP) measurements, which are one of the most important characteristics for the determination of stability, were performed in a pH range from 3 to 8 (Fig. 2). In the case of ZnS particles dispersed in distilled water (ZnS-H_2_O), it can be evidenced that the sample reached positive values of ZP in almost entire studied pH range. These obtained values are due to the positive Zn(II) ions present at the surface of the particles and their transfer into the water. The highest value of ZP was obtained at pH 3 (+19 mV). With the increasing pH, the ZP reached less positive values and the isoelectric point (IEP) of ZnS nanoparticles was determined at pH 7.3. Our value is considerably higher in comparison with the literature sources, where the IEPs were referred below 3.0 [26], or in the case of natural sphalerite (ZnS) at 3.0 [27], or for synthetic ZnS prepared by [28], the values in a range 3.0–3.5 were obtained. This increase is connected with the high specific surface area (126 m^2^/g [13]) of the mechanochemically prepared ZnS and subsequently higher amount of active sites, which are available for the dissolution of Zn(II) ions from the surface. During the mechanochemical synthesis, a lot of defects, cracks, open pores, and intergranular spaces are created at the surface of the samples [29], and in many cases, such samples allocate the increased reactivity [30–32].

After the milling of ZnS sample in chitosan solution (ZnS-chitosan), the increase in the ZP to more positive values was evidenced in the entire studied pH range. This shift was caused by properties of chitosan. Chitosan is a cationic polymer, with pKa ~6.5, which is insoluble in water at neutral pH, at which the majority of amines from the molecule are deprotonated. On the other hand, at acidic pH, the chitosan becomes water soluble, as it is positively charged [33]. The creation of ZnS-chitosan colloidal system brought about the increased stability of the suspension from incipient instability area to good stability area (up to +57 mV at pH 3). As was determined earlier [17, 21, 34–36], Zn(II) ions presented at the surface of particles can interact with the amine, amide, and hydroxyl groups of chitosan. As a consequence, the coating of ZnS nanocrystals by chitosan has led to high positive values of ZP and to their better stability.

**Fig. 3:**
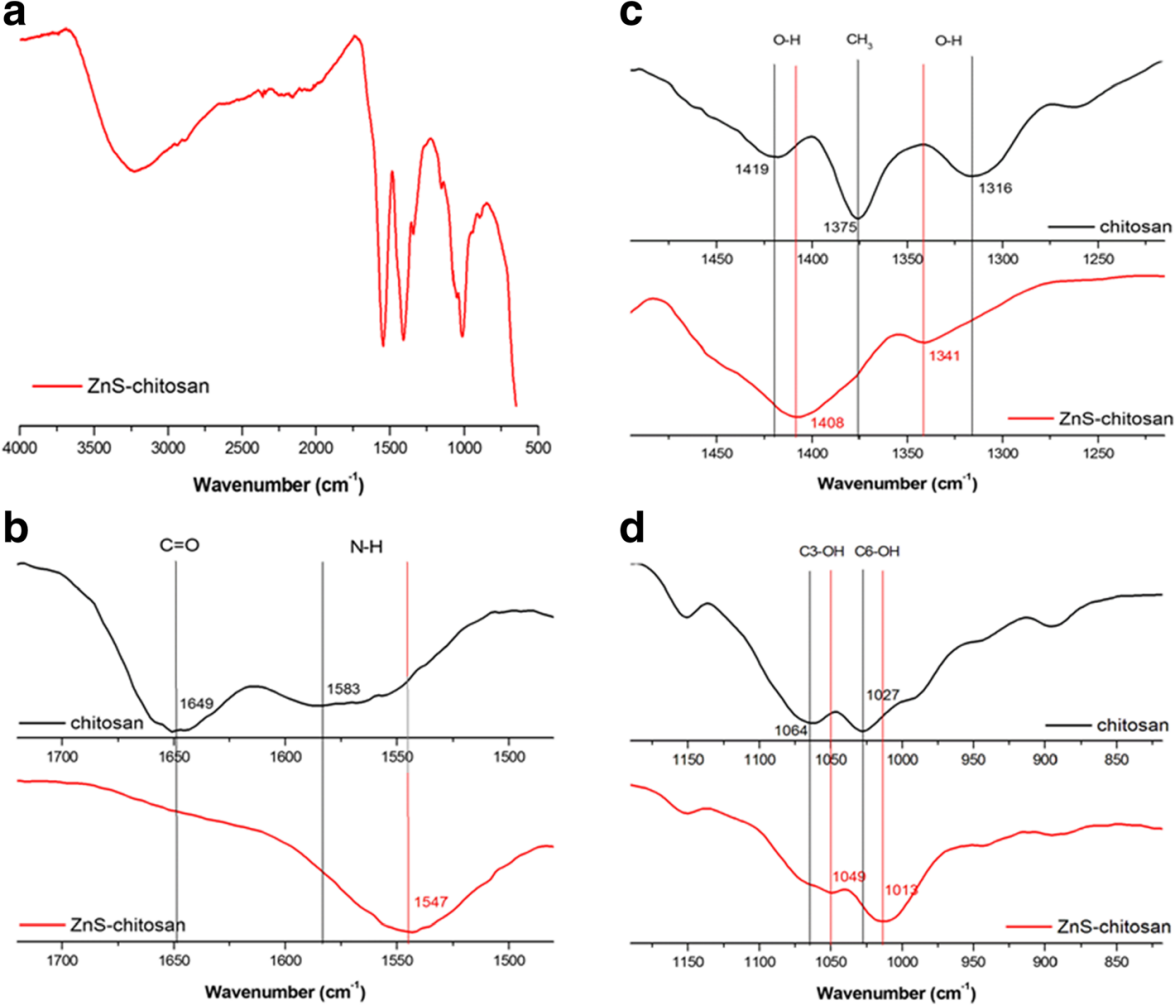
FTIR spectra of **a** chitosan-coated ZnS, **b** amide I and II band, **c** hydroxyl group in a ring, and **d** secondary and primary alcohol vibrations. *Black lines* vibrations for pure chitosan, *red lines* vibrations for chitosan-coated ZnS nanocrystals

The possible interaction between ZnS particles and chitosan were studied using FTIR spectroscopy. The vibrations of pure chitosan were described in detail in our previous work [17]. As was mentioned in that paper, amine, amide, and hydroxyl groups are the most reactive sites of chitosan and are involved in the interactions with the ambient cations and anions. In Fig. 3a, the spectrum of chitosan-coated ZnS nanocrystals is shown. Some changes in comparison with pure chitosan spectrum can be noticed. The individual groups, which were involved in the interactions, are illustrated in Fig. 3b–d. The visible shifts of vibrations occurred mainly in the amide I band corresponding to the carbonyl C=O stretching of the amide group and amide II band corresponding to the N–H bending vibrations in amide group and of the deacetylated primary amine –NH_2_ (shifts from 1649 and 1583 cm^−1^ to 1547 cm^−1^, respectively, Fig. 3b). Further changes are denoted in the hydroxyl group attributed to the OH and CH vibrations present in the ring (from 1419 and 1316 cm^−1^ to 1408 and 1341 cm^−1^, respectively, Fig. 3c) and in the secondary and primary alcohol vibrations (from 1064 cm^−1^ for C3–OH vibration in the secondary alcohol and 1027 cm^−1^ for C6–OH vibration in the primary alcohol to 1049 and 1013 cm^−1^, respectively, Fig. 3d). The similar shifts were observed in a binary InAs/ZnS nanocomposite system prepared in chitosan [17]. These shifts indicate that interaction between Zn(II) ions and chitosan indeed exist.

### Optical Properties of Chitosan-Coated ZnS Nanocrystals

**Fig. 4:**
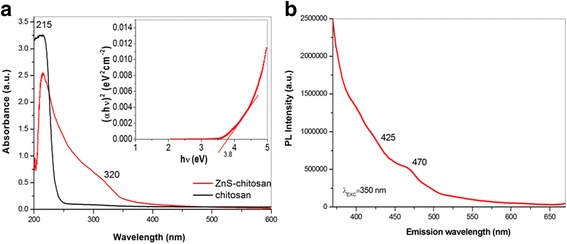
**a** UV–Vis spectra of pure chitosan (*black curve*) and chitosan-coated ZnS nanocrystals (*red curve*); *inset*: Tauc relation. **b** PL spectrum of chitosan-coated ZnS nanocrystals

The optical properties of chitosan-coated ZnS nanocrystals were investigated using UV–Vis and PL spectroscopy measurements. Appropriate UV–Vis and PL spectra are shown in Fig. 4a, b.

The characteristic absorption peak for chitosan below 220 nm [37] can be also seen in our sample located at 215 nm (5.7 eV) (Fig. 4a). It is in good accordance with the result present in our previous paper [17]. The absorption peak observed at 320 nm (3.8 eV) for chitosan-coated ZnS nanocrystals is strongly blue shifted with respect to the bulk ZnS reported at 340 nm (3.6 eV) [21]. The higher optical bandgap observed for our sample is likely due to the well-known quantum confinement effect [38]. The observed absorption peaks indicate the existence of a chemical bond between chitosan and ZnS nanocrystals [39].

The bandgap of chitosan-coated ZnS nanocrystals was approximated using the Tauc relation [40] extracted from the UV–Vis spectrum, considering ZnS as a direct bandgap semiconductor, by plotting the squared absorbance versus energy and extrapolating to zero, as shown in the inset of Fig. 4a. The bandgap of chitosan-coated ZnS nanocrystals is estimated to be 3.8 eV, which is in good agreement with the previous reports [21, 41] and is assigned to the optical transitions of the excitonic states in ZnS. The obvious blue shift could be attributed to the existence of very small ZnS nanocrystalline particles [38].

The emission spectrum was recorded at excitation wavelength 350 nm as shown in Fig. 4b. However, in the majority of the previous papers, rather than the band-edge emission in the UV wavelength range, ZnS nanocrystals always exhibit radiative recombination in the wavelength range of 400–550 nm at room temperature which is related to surface states or deep-level defects [42–44]. A very weak PL peak of chitosan-coated ZnS nanocrystals is centered at 425 nm (2.9 eV), and a little stronger one is located at 470 nm (2.6 eV). The emission bands below 450 nm are mostly associated with *V*_s_ (vacancies of sulfur, S^2−^) and *I*_Zn_ (Zn^2+^ at interstitial sites at the nanocrystal lattice) defects, and the band at 470 nm may be assigned to surface defects according to the energy-level diagrams described by Wageh [45].

### In Vitro Studies

**Fig. 5:**
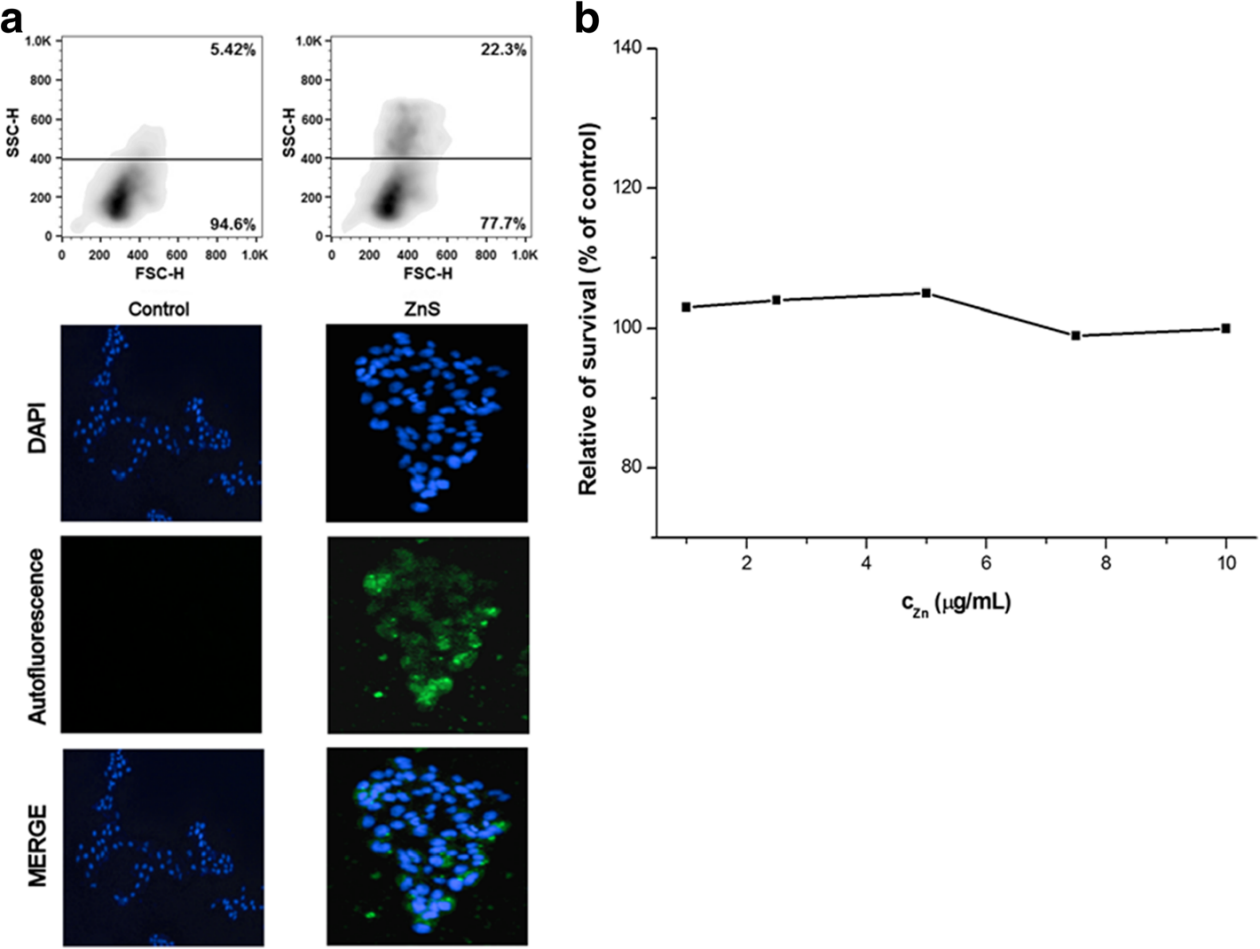
**a** Flow cytometry and fluorescence microscopy analysis and **b** relative survival of CaCo-2 cells after their treatment with chitosan-coated ZnS nanocrystals

**Fig. 6:**
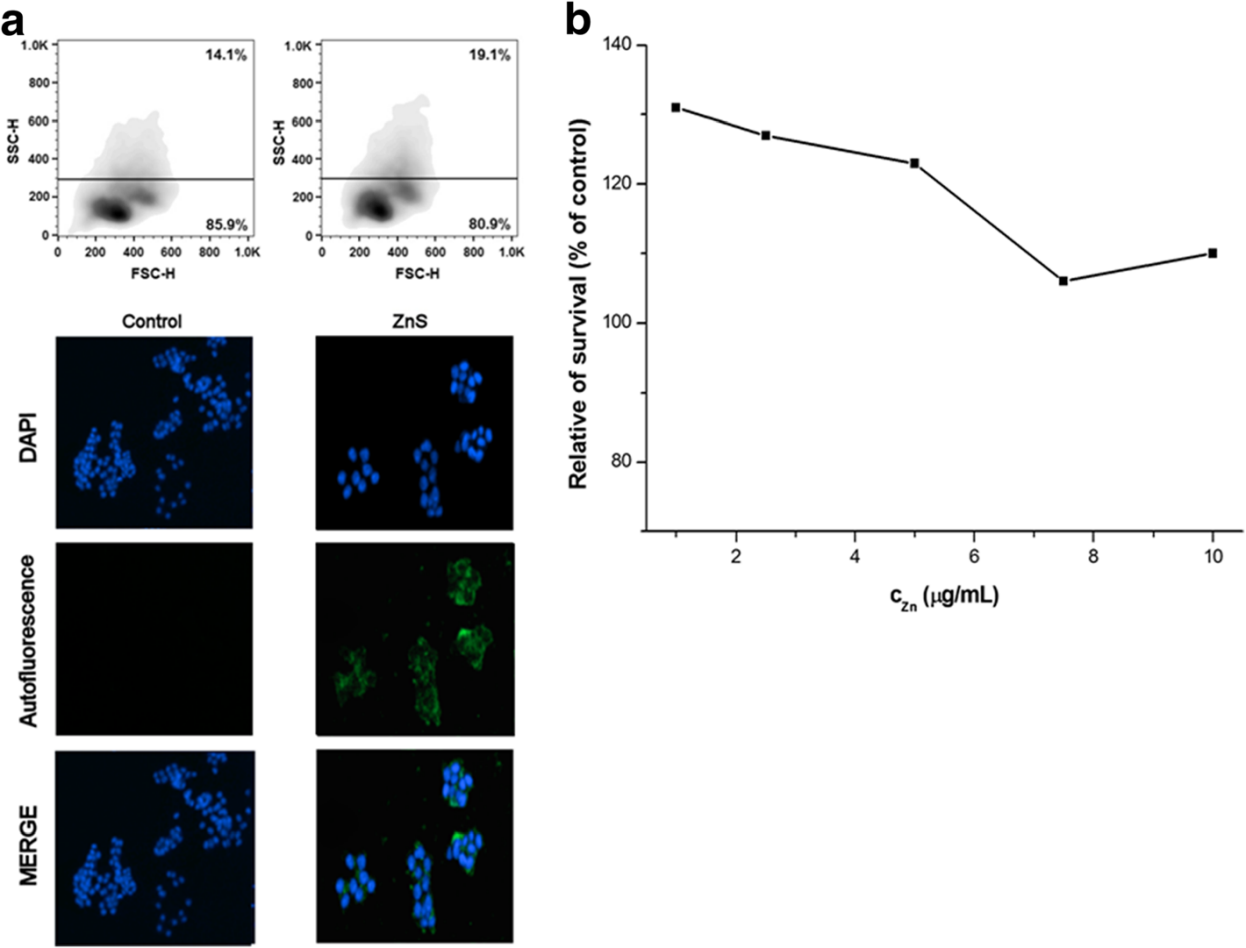
**a** Flow cytometry and fluorescence microscopy analysis and **b** relative survival of HCT116 cells after their treatment with chitosan-coated ZnS nanocrystals

**Fig. 7:**
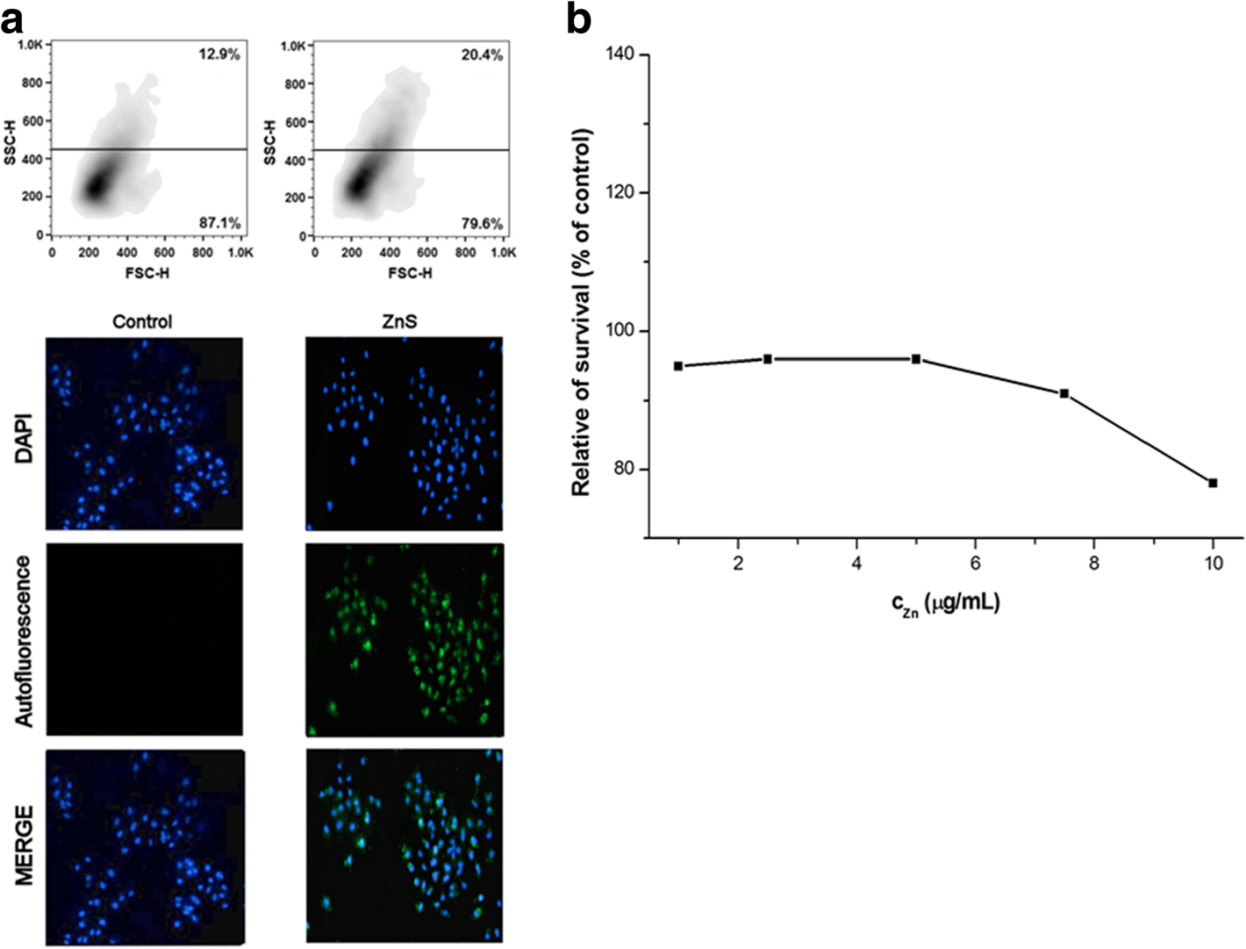
**a** Flow cytometry and fluorescence microscopy analysis and **b** relative survival of HeLa cells after their treatment with chitosan-coated ZnS nanocrystals

**Fig. 8:**
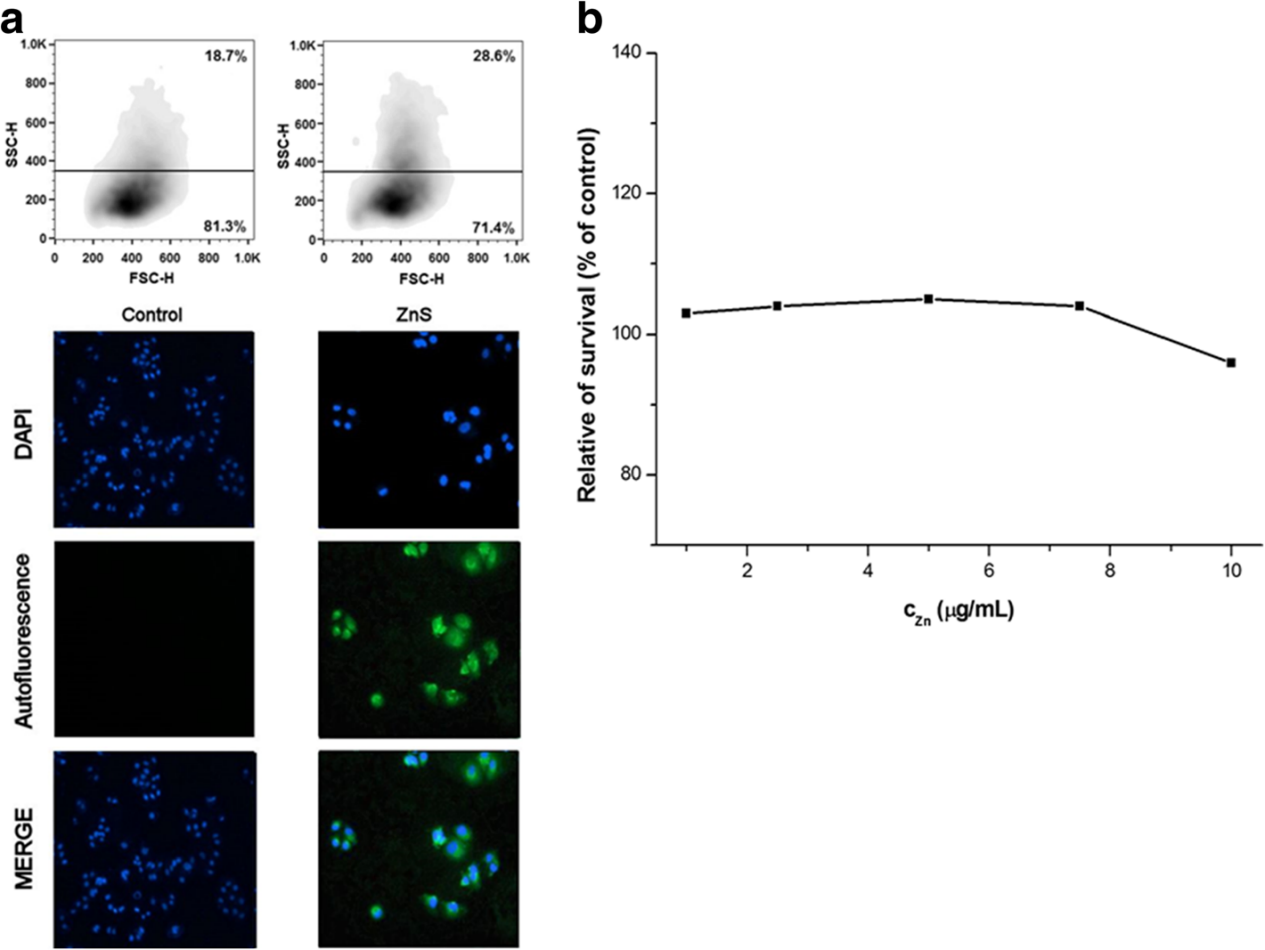
**a** Flow cytometry and fluorescence microscopy analysis and **b** relative survival of MCF-7 cells after their treatment with chitosan-coated ZnS nanocrystals

For in vitro tests of chitosan-coated ZnS nanocrystals, four cancer cell lines, CaCo-2, HCT116, HeLa, and MCF-7, have been applied. For the studying of nanocrystal behavior in these cell lines, fluorescence microscopy and flow cytometry analysis showing granularity were applied (Figs. 5, 6, 7, and 8a). The cancer cells were cultivated with ZnS nanocrystals (*c*_Zn_ = 0.5 μg/mL) for 72 h. For the live cell imaging analysis, cell nuclei were stained with DAPI and the images of nanocrystal autofluorescence were acquired sequentially and then combined using Gene5 software (merge).

From the microscopic point of view, it can be seen that the fluorescent nanocrystals passed through the cell membrane, entered into the cytoplasm, and surrounded the nucleus (Figs. 5, 6, 7, and 8a bottom). In many cells, the nucleus was observed as a distinct object with nanocrystals outlining it as is shown on the merged pictures. Similar observations were found also in the case of BaTiO_3_ nanoparticles [46].

According to flow cytometry analysis, namely forward and side scatter of light (FSC-H and SSC-H), which are proportional to cell size and to their granularity, respectively, some changes in these characteristics can be seen, when applying chitosan-coated ZnS nanocrystals (Figs. 5, 6, 7, and 8a top). The granularity of cells was significantly increased in all the cases as a consequence of cellular uptake of these particles into the cytoplasm. On the other hand, the changes in the cell sizes were not evident. Similar but stronger effect was observed in our previous research after the treatment of cells with the chitosan-coated InAs/ZnS nanocrystals, where not only the granularity of cancer cells was rapidly increased but also they were enlarged (mostly in the case of CaCo-2 and HeLa cell lines) [17].

The metabolic activity of four cancer cell lines after their exposition toward the ZnS nanocrystals was also pursued. The results are depicted in Figs. 5, 6, 7, and 8b. From the obtained results, it can be clearly seen that the ZnS nanocrystals do not influence the metabolic activity of the studied cancer cell lines. Only in the case of HeLa cell line, the survival of cells was decreased to 80% when applying the highest studied concentration of zinc (10 μg/mL). According to the observed results, it can be concluded that the studied suspension is not toxic and it has fluorescence properties, which could be used in bio-imaging applications.

## Conclusions

In this paper, the nanosuspension of chitosan-coated zinc sulfide nanocrystals was prepared by wet ultra-fine milling process for the first time. The nanosuspension was very stable, and the zeta potential reached high positive values (up to +57 mV). As a consequence, the nanoparticles in the suspension did not agglomerate and settle down. Using FTIR spectroscopy, it was found that the amine, amide, and hydroxyl groups from chitosan were actively involved in bonding with Zn(II) ions from ZnS. The promising results from measurements of optical properties (using UV–Vis and PL spectroscopy) were verified on four different cancer cell lines, and the autofluorescence of the prepared nanocrystals was evidenced. The cells were more visible in comparison with non-treated ones under the fluorescence microscope. Moreover, chitosan-coated ZnS nanocrystals suggest to be nontoxic, and the nanoparticles did not influence the metabolism of the cells. According to the results of the present study, as well as to that mentioned in the literature, it seems that the ZnS nanocrystals could be used as alternative to conventional imaging agents.

## Abbreviations

FSC-H: Forward scatter of light
FTIR: Fourier transform infrared
IEP: Isoelectric point
MTS: Metabolic activity colorimetric assay
PL: Photoluminescence
SSC-H: Side scatter of light
UV–Vis: Ultraviolet-visible
ZP: Zeta potential

## References

[1] Caruso F (2001). Nanoengineering of particle surfaces. Adv Mater.

[2] Dharshini MP, Shally V, Jayam SG, Delphine SM (2014). Improved optical properties of sulphide based core/shell nanostructures. J Appl Phys.

[3] Mathew ME, Mohan JC, Manzoor K, Nair SV, Tamura H, Jayakumar R (2010). Folate conjugated carboxymethyl chitosan-manganese doped zinc sulphide nanoparticles for targeted drug delivery and imaging of cancer cells. Carbohydr Polym.

[4] Ahmed KBA, Ahalya P, Sengan M, Kamlekar R, Verappan A (2015). Synthesis and characterization of zinc sulphide quantum dots and their interaction with snake gourd (Trichsantes angiuna) seed lectin. Spectrochim Acta A.

[5] Sharma P, Brown S, Walter G, Santra S, Moudgil B (2006). Nanoparticles for bioimaging. Adv Colloid Interface Sci.

[6] Chatterjee K, Sarkar S, Rao KJ, Paria S (2014). Core/shell nanoparticles in biomedical applications. Adv Colloid Interface Sci.

[7] Geszke-Moritz M, Moritz M (2013). Quantum dots as versatile probes in medical sciences: synthesis, modification and properties. Mater Sci Eng C.

[8] Nakatsuka N, Barnaby SN, Tsiola A, Fath KR, Williams BA, Banerjee IA (2013). Self-assembling peptide assemblies bound to ZnS nanoparticles and their interactions with mammalian cells. Colloids Surf B.

[9] Bujnakova Z, Dutkova E, Balaz M, Turianicova E, Balaz P (2015). Stability studies of As_4_S_4_ nanosuspension prepared by wet milling in Poloxamer 407. Int J Pharm.

[10] Chen H, Yuan L, Song W, Wu ZK, Li D (2008). Biocompatible polymer materials: role of protein-surface interactions. Prog Polym Sci.

[11] Bujnakova Z, Balaz P, Makreski P, Jovanovski G, Caplovicova M, Caplovic L (2015). Arsenic sulfide nanoparticles prepared by milling: properties, free-volume characterization, and anti-cancer effects. J Mater Sci.

[12] Helgason T, Awad TS, Kristbergsson K, McClements DJ, Weiss J (2009). Effect of surfactant surface coverage on formation of solid lipid nanoparticles (SLN). J Colloid Interface Sci.

[13] Bujnakova Z, Balaz M, Zduriencikova M, Sedlak J, Caplovicova M, Caplovic L (2017). Preparation, properties and anticancer effects of mixed As_4_S_4_/ZnS nanoparticles capped by Poloxamer 407. Mater Sci Eng C.

[14] Sarno M, Cirillo C, Ciambelli P (2015). Fluorescent and magnetic monodisperse Fe_3_O_4_ nanoparticles. Chem Eng Trans.

[15] Li Z, Liangfang Z (2010). Lipid-polymer hybrid nanoparticles: synthesis, characterization and applications. Nano LIFE.

[16] Bujnakova Z, Balaz M, Dutkova E, Balaz P, Kello M, Mojzisova G (2017). Mechanochemical approach for the capping of mixed core CdS/ZnS nanocrystals: elimination of cadmium toxicity. J Colloid Interface Sci.

[17] Bujnakova Z, Dutkova E, Zorkovska A, Balaz M, Kováč J, Kello M (2017). Mechanochemical synthesis and in vitro studies of chitosan-coated InAs/ZnS mixed nanocrystals. J Mater Sci.

[18] Rampino A, Borgogna M, Blasi P, Bellich B, Cesaro A (2013). Chitosan nanoparticles: preparation, size evolution and stability. Int J Pharm.

[19] Jayakumar R, Prabaharan M, Muzzarelli RAA (2011). Chitosan for biomaterials I.

[20] Chang SQ, Kang B, Dai YD, Zhang HX, Chen D (2011). One-step fabrication of biocompatible chitosan-coated ZnS and ZnS:Mn^2+^ quantum dots via a gamma-radiation route. Nanoscale Res Lett.

[21] Ramanery FP, Mansur AAP, Mansur HS (2013). One-step colloidal synthesis of biocompatible water-soluble ZnS quantum dot/chitosan nanoconjugates. Nanoscale Res Lett.

[22] Augustine MS, Anas A, Das AV, Sreekanth S, Jayalekshmi S (2015). Cytotoxicity and cellular uptake of ZnS:Mn nanocrystals biofunctionalized with chitosan and amino acids. Spectrochim Acta A.

[23] Balaz P, Boldizarova E, Godocikova E, Briacin J (2003). Mechanochemical route for sulphide nanoparticles preparation. Mater Lett.

[24] Dutkova E, Balaz P, Pourghahramani P, Velumani S, Ascencio JA, Kostova NG (2009). Properties of mechanochemically synthesized ZnS nanoparticles. J Nanosci Nanotechnol.

[25] Balaz P, Balaz M, Dutkova E, Zorkovska A, Kovac J, Hronec P (2016). CdS/ZnS nanocomposites: from mechanochemical synthesis to cytotoxicity issues. Mater Sci Eng C.

[26] Liu JC, Huang CP (1992). Electrokinetic characteristics of some metal sulfide water interfaces. Langmuir.

[27] Pugh RJ, Tjus K (1987). Electrokinetic studies on Cu(Ii) hydroxy coated zinc-sulfide particles. J Colloid Interface Sci.

[28] Williams R, Labib ME (1985). Zinc-sulfide surface-chemistry—an electrokinetic study. J Colloid Interface Sci.

[29] Dutkova E, Balaz P, Pourghahramani P, Balek V, Nguyen AV, Satka A (2012). Mechanochemically synthesised ZnxCd1-xS nanoparticles for solar energy applications. J Nano Res.

[30] Wieczorek-Ciurowa K, Gamrat K (2007). Some aspects of mechanochemical reactions. Materials Science-Poland.

[31] Balaz P, Pourghahramani P, Achimovicova M, Dutkova E, Kovac J, Satka A (2011). Mechanochemical synthesis and reactivity of PbS nanocrystals. J Cryst Growth.

[32] Balaz P, Achimovicova M, Balaz M, Billik P, Cherkezova-Zheleva Z, Criado JM (2013). Hallmarks of mechanochemistry: from nanoparticles to technology. Chem Soc Rev.

[33] Bowman K, Leong KW (2006). Chitosan nanoparticles for oral drug and gene delivery. Int J Nanomed.

[34] Pawlak A, Mucha A (2003). Thermogravimetric and FTIR studies of chitosan blends. Thermochim Acta.

[35] Bhattarai SR, Kc RB, Kim SY, Sharma M, Khil MS, Hwang PH (2008). N-hexanoyl chitosan stabilized magnetic nanoparticles: implication for cellular labeling and magnetic resonance imaging. J Nanobiotechnology.

[36] Salehizadeh H, Hekmatian E, Sadeghi M, Kennedy K (2012). Synthesis and characterization of core-shell Fe_3_O_4_-gold-chitosan nanostructure. J Nanobiotechnology.

[37] Kumirska J, Czerwicka M, Kaczynski Z, Bychowska A, Brzozowski K, Thoming J (2010). Application of spectroscopic methods for structural analysis of chitin and chitosan. Mar Drugs.

[38] Brus LE (1984). Electron-electron and electron-hole interactions in small semiconductor crystallites: the size dependence of the lowest excited electronic state. J Chem Phys.

[39] Viswanath R, Naik HSB, Somalanaik YKG, Neelanjeneallu PKP, Harish KN, Prabhakara MC (2014). Studies on characterization, optical absorption, and photoluminescene of yttrium doped ZnS nanoparticles. J Nanotechnol.

[40] Tauc J, Menth A (1972). States in the gap. J Non-Cryst Solids.

[41] Cao XY, Shen F, Zhang MW, Bie JX, Liu X, Luo YL (2014). Facile synthesis of chitosan-capped ZnS quantum dots as an eco-friendly fluorescence sensor for rapid determination of bisphenol A in water and plastic samples. RSC Adv.

[42] Mansur HS, Mansur AAP, Ramanery FP, Borsagli FGLM (2014). Green and facile synthesis of water-soluble ZnS quantum dots nanohybrids using chitosan derivate ligands. J Nanopart Res.

[43] Wang YW, Zhang LD, Liang CH, Wang GZ, Peng XS (2002). Catalytic growth and photoluminescence properties of semiconductor single-crystal ZnS nanowires. Chem Phys Lett.

[44] Mansur HS, Mansur AAP, Soriano-Araujo A, Lobato ZIP (2015). Beyond biocompatibility: an approach for the synthesis of ZnS quantum dot-chitosan nano-immunoconjugates for cancer diagnosis. Green Chem.

[45] Wageh S, Ling ZS, Xu-Rong X (2003). Growth and optical properties of colloidal ZnS nanoparticles. J Cryst Growth.

[46] Wang SW, Zhao XY, Qian J, He SL (2016). Polyelectrolyte coated BaTiO3 nanoparticles for second harmonic generation imaging-guided photodynamic therapy with improved stability and enhanced cellular uptake. RSC Adv.

